# Otite externe maligne à Candida Albicans

**DOI:** 10.11604/pamj.2016.24.322.6007

**Published:** 2016-08-22

**Authors:** Fahd Elayoubi, Azeddine Lachkar, Ahmed Aabach, Mohamed Chouai, Mohamed Rachid Ghailan

**Affiliations:** 1 Service d’Oto-Rhino-Laryngologie et Chirurgie Cervico-Faciale CHU Mohammed VI, Oujda, Maroc

**Keywords:** Otite externe maligne, Candida albicans, examen mycosique, Malignant otitis externa, Candida albicans, fungal examination

## Abstract

L’otite externe maligne est une ostéomyélite de la base du crane. Le Pseudomonas aeruginosa est le germe le plus incriminé. Cependant l’origine fongique n’est pas rare. Patiente âgée de 80 ans avait présenté une otalgie gauche persistante depuis deux mois malgré un traitement bien conduit. L’examen otologique mettait en évidence des signes inflammatoires au niveau du pavillon, une sténose du conduit avec des granulomes, et otorrhée d’allure purulente. Le scanner montrait un comblement otomastoïdien, un processus inflammatoire extensif des tissus pré et rétro-auriculaire et une lyse du tympanal. Vu l’absence d’amélioration un examen mycologique a été réalisé et qui a révélé la présence de Candida Albicans. Les cas d’otite externe maligne à Candida Albicans sont rarement rapportés. L’origine fongique doit être suspecté devant la négativité des prélèvements bactériologiques et la non amélioration malgré un traitement antibiotique bien conduit, et confirmée par des prélèvements mycologiques parfois multiples. L’otite externe maligne à Candida Albicans est une infection rare potentiellement mortelle.

## Abstract (en)

Malignant otitis externa is also referred to as skull base osteomyelitis. Pseudomonas aeruginosa is the most common offending pathogen. However, fungal origin is not uncommon. 80-year-old patient having persistent left earache since two months despite adequate treatment. Otologic examination showed signs of inflammation in the auricular pavilion, ear canal stenosis with granulomas and purulent otorrhea. CT scan showed a filled otomastoid, extensive inflammatory process affecting the pre-auricular and retro-auricular tissues and lysis of the tympanic bulla. Given the lack of improvement, mycological examination was performed which revealed the presence of Candida Albicans. Cases of malignant otitis externa caused by Candida albicans are rarely reported. Fungal origin should be suspected in patients who have negative bacteriological samples and no improvement despite adequate antibiotic treatment. It should be confirmed by sometimes multiple mycological samples. Malignant otitis externa caused by Candida albicans is a rare potentially mortal infection.

## Introduction

En 1959 Melter et Kelemen décrivent le premier cas d'otite externe compliquée d'une ostéomyélite de la base du crane, mais se sont les travaux de Chandler en 1968 qui ont permis de définir cette infection et lui attribuer le terme d'otite externe maligne du fait de sa gravité et de son évolution souvent fatale [1]. Au cours de ces dernières années plusieurs travaux sur l'otite externe maligne ont été publiés, le Pseudomonas aeruginosa était le germe le plus incriminé. Cependant ce concept d'otite externe maligne à Pseudomonas aeruginosa chez un patient diabétique n'est plus la règle [2]. Le but de ce travail est d'illustrer sur la base d'un cas, une otite externe maligne à Candida Albicans chez une patiente diabétique.

## Patient et observation

**Figure 1:**
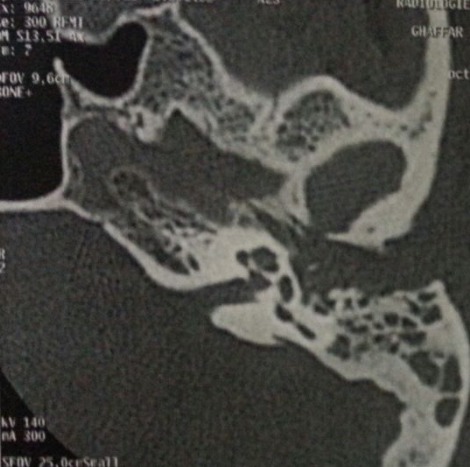
Scanner des rochers en coupe axiale montrant un comblement du méat acoustique externe et otomastoïdien avec lyse du tympanal

**Figure 2:**
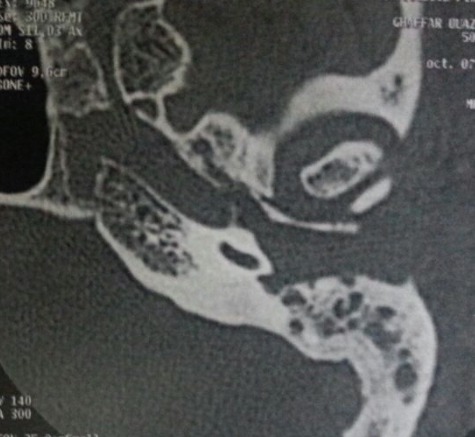
Scanner du rocher gauche: comblement otomastoïdien et lyse de la mastoïde

Il s'agit d'une patiente âgée de 80 ans. Dans ses antécédents on note un diabète depuis 20 ans sous insuline, ainsi qu'un important terrain vasculaire (artériopathie des membres inférieurs, une hypertension artérielle traitée, et une dyslipidémie). Elle avait présenté une otalgie gauche persistante depuis deux mois. Le diagnostic retenu était celui d'otite externe et le traitement préconisé des antibiotiques et des corticoïdes par voie topique. Un mois après, la douleur persistait avec apparition de céphalées, ce qui a motivé le transfert de la patiente dans notre structure. L'examen otologique mettait en évidence des signes inflammatoires au niveau du pavillon, une sténose du conduit avec des granulomes, et otorrhée d'allure purulente, par ailleurs le tympan était non vu. Le scanner montrait un comblement otomastoïdien, un processus inflammatoire extensif des tissus pré et rétro-auriculaire et une lyse du tympanal (Figure 1, Figure 2). Le diagnostic d'otite externe maligne avait été posé et une antibiothérapie injectable empirique instaurée à base de céphalosporines de troisième génération et de ciprofloxacine, la mise en place de Pop-Otowick, ainsi que des soins réguliers de l'oreille (nettoyage, élimination des débris). La recherche bactériologique était négative. Une biopsie du conduit montrait un granulome inflammatoire. Vu l'absence d'amélioration après 25 jours d'antibiothérapie, un examen mycologique a été réalisé et qui a révélé la présence de Candida Albicans. Le diagnostic d'otite externe maligne fongique a été retenu et le traitement a consisté en de l'amphotéricine B par voie intraveineuse pendant 2 semaines avec relais par l'itraconazole par voie orale pendant 10 semaines. L'évolution clinique était favorable avec disparition de la symptomatologie douloureuse et régression des anomalies otoscopiques en 3 semaines.

## Discussion

La description classique d'un patient diabétique souffrant d'une otite externe maligne à Pseudomonas Aeruginosa est probablement vraie pour la plupart des cas, mais certainement pas pour tous, comme l'illustre le cas présenté dans ce travail. Le Pseudomonas Aeruginosa est le germe le plus incriminé, responsable de plus de 95% des cas. Toutefois d'autres bactéries en sont parfois la cause, comme le Staphylococcus aureus, ou epidermidis, le Proteus mirabilis, la Klebsiella oxytoca, ainsi que des champignons tels le Candida ciferri ou parapsilosis, le Malassezia sympodialis, le Scedosporium apiospermum, ou l'Aspergillus fumigatus ou niger [3]. Les cas d'otite externe maligne à Candida Albicans sont rarement rapportés. L'origine fongique doit être suspectée devant la négativité des prélèvements bactériologiques et la non amélioration malgré un traitement antibiotique bien conduit, et confirmée par des prélèvement mycologiques parfois multiples. L'imagerie est certainement utile pour un bilan d'extension des lésions. Toutefois l'importance des lésions décelables par le scanner n'est pas un facteur pronostique, l'imagerie n'est pas non plus l'élément déterminent pour le suivi de la maladie et l'évaluation de la réponse au traitement [4]. La scintigraphie au technétium 99m permet d'établir le diagnostic à un stade précoce avant que les lésions de déminéralisation osseuse ne deviennent radiologiquement décelables. La scintigraphie au Gallium 67 est indiquée dans le suivi thérapeutique, elle confirme la guérison et ainsi l'arrêt du traitement [5]. Le traitement de l'otite externe maligne fongique est essentiellement médical et doit être instauré le plus rapidement possible. Il fait appel à l'amphotéricine B par voie intraveineuse et à l'itraconazole par voie orale, ce traitement doit être prolongé pendant 3 à 6 mois. Le traitement local est indispensable et consiste en des soins locaux quotidiens avec nettoyage et calibrage du conduit, exérèse des séquestres et tissus nécrosés. Le contrôle du diabète est obligatoire. La mortalité liée à l'otite externe maligne fongique n'est pas évaluée vu le peu de séries retrouvée dans la littérature [6].

## Conclusion

L'otite externe maligne à Candida Albicans est une infection rare potentiellement mortelle. Elle doit être évoquée devant l'absence d'amélioration sous traitement antibiotique. Aussi une ostéolyse sur une otite externe doit conduire à une recherche fongique car le diagnostic précoce de cette affection est associé à une chance accrue de succès thérapeutique.
